# The consequences of Ireland’s culture of medical migration

**DOI:** 10.1186/s12960-017-0263-7

**Published:** 2017-12-28

**Authors:** Niamh Humphries, Sophie Crowe, Cian McDermott, Sara McAleese, Ruairi Brugha

**Affiliations:** 1 Research Department, Royal College of Physicians of Ireland, Dublin, Ireland; 2 Department of Epidemiology and Public Health Medicine, Royal College of Surgeons in Ireland, Dublin, Ireland; 3 University Hospital Geelong, Barwon Health, Geelong, Victoria Australia

**Keywords:** Medical workforce, Medical migration, Health system research, Culture, Qualitative research, Ireland

## Abstract

**Background:**

In recent years, Ireland has experienced a large-scale, outward migration of doctors. This presents a challenge for national policy makers and workforce planners seeking to build a self-sufficient medical workforce that trains and retains enough doctors to meet demand. Although, traditionally, medical migration has been considered beneficial to the Irish health system, austerity has brought a greater level of uncertainty to the health system and, with it, a need to reappraise the professional culture of migration and its impact on the Irish health system.

**Methods:**

This paper illustrates how a culture of migration informs career and migration plans. It draws on quantitative data—registration and migration data from source and destination countries—and qualitative data—in-depth interviews with 50 doctors who had undertaken postgraduate medical training in Ireland.

**Results:**

Of 50 respondents, 42 highlighted the importance of migration. The culture of medical migration rests on two assumptions—that international training/experience is beneficial to all doctors and that those who emigrate will return to Ireland with additional skills and experience. This assumption of return is challenged by a new generation of doctors whose professional lives have been shaped by globalisation and by austerity. Global comparisons reveal the comparatively poor working conditions, training and career opportunities in Ireland and the relative attractiveness of a permanent career abroad.

**Conclusion:**

In light of these changes, there is a need to critically appraise the culture of medical migration to determine if and in what circumstances migration is appropriate to the needs of the Irish health system. The paper considers the need to reappraise the culture of medical migration and the widespread emigration that it promotes.

## Background

Medical migration involves the movement of a qualified doctor from one country to another to practice medicine. It includes those who commute across borders and those who migrate temporarily to obtain specialist training, as well as those who migrate on a permanent basis [1]. As the definition implies, medical migration impacts upon individuals (and their families), the medical profession and the national health systems of both source and destination countries. At each of these levels, the free movement of doctors has advantages—providing an opportunity for doctors to obtain new skills, to enhance their careers and/or their earning potential and to provide a better future for themselves and their families. In facilitating the sharing of skills and knowledge, medical migration can advance medical research and improve practice. At a national level, medical migration can reduce doctor unemployment and/or underemployment and generate a valuable remittance flow to the source country. However, medical migration also presents challenges at each of these levels, one of which is that when doctors migrate from a high-income country in large numbers, vacancies are created which attracts doctors from low- and middle-income countries (LMIC).

The Global Strategy for Human Resources for Health acknowledges the potential benefits of international migration, while also highlighting the importance of retention [2] to the delivery of healthcare. For a health system to function, it must train and retain enough doctors to deliver care. This paper focusses on the consequences of Ireland’s professional culture of medical migration, both for Ireland and for its global obligations. The global significance of this as a research topic stems from the fact that Ireland’s failure to retain doctors has translated into heavy reliance on internationally trained doctors to staff the Irish health system [3]. Internationally trained doctors now account for 38% of all registered doctors [4] and 77% of non-trainee junior hospital doctors in Ireland [4]. In this way, the local issue of doctor emigration from a high-income source country (Ireland) quickly becomes a global issue, as Ireland recruits many of its internationally trained doctors from LMICs, such as Pakistan, India, Sudan and Nigeria) [5].

In recognition of the fact that national skill shortages can cause further imbalances at a global level, the WHO Global Code on the International Recruitment of Health Personnel calls on high-income countries, such as Ireland, to strive for self-sufficiency, that is to ‘educate, retain and sustain’ [6] enough doctors to staff their health systems. Similarly, the Global Strategy on Human Resources for Health calls for a ‘decrease[d] reliance on foreign trained doctors’ [2]. To achieve a self-sufficient medical workforce, it is essential that Ireland identifies and addresses the drivers of medical migration.

This paper draws on qualitative data generated from in-depth interviews with 50 doctors who had undertaken postgraduate medical training in Ireland, as well as registration migration data on doctor migration from Ireland to five key destination countries. It illustrates how a professional culture of migration can inform career decision making and considers how this might influence migration patterns. The authors speculate that a strong professional culture of migration, combined with an era of ‘greater global connectivity’ [7] and difficult working conditions in the Irish health system [3], has created optimal conditions for ongoing large-scale doctor emigration. In his analysis of the health worker migration from Pacific island states, Connell emphasised that policies seeking to slow or discourage doctor migration must confront these cultures of migration [8]. This paper seeks to initiate debate in Ireland and other high-income countries about the clash between the professional culture of migration and the policy objective of medical workforce self-sufficiency.

## Culture of medical migration

Push/pull analyses have dominated the literature on health worker migration [9–12]. Although important components of doctor migration, they cannot fully explain the complexities of migrant decision making. Political, economic [10] and cultural contexts [8] are also significant. Connell has highlighted the potential impact of migration cultures—national and professional—on health worker migration [8].

In some low- and middle-income countries (LMICs), migration is regarded as an essential component of the successful medical career—practicing medicine and ‘training outside the. . . home country is superior and a mark of achievement’ [13]*.* In such circumstances, migration is considered essential to career progression. In this paper, the authors contend that in Ireland—a high-income country—a similar culture of medical migration and a professional bias towards international experience is displayed. The senior doctors who preside over interview and promotion panels—the gatekeepers of the profession [14]—appear to give preferential treatment to doctors with international experience. In this way, migration is presented as an essential (rather than an optional) component of a successful medical career, and this is conveyed to a new generation of doctors via recruitment and promotion decisions. The professional culture of medical migration encourages rather than discourages doctors to emigrate, considering it acceptable and necessary, rather than problematic [8].

## Medical migration

Today’s migrant doctors migrate from Ireland for postgraduate training, for international experience, to access better working conditions [3, 15] and sometimes as *backpacker migrants* [1] taking a time-out after completing internship and before beginning specialist training. Doctors are highly skilled migrants, who are in demand globally. Those who have trained or practiced in Ireland are native or fluent English speakers, further enhancing their attractiveness as migrants to English-speaking destination countries such as Australia, the UK, the USA, Canada and New Zealand. The ‘migration aspirations of young people today. . . are not just a replica...of previous generations’ [7]. Their global connectivity [7] enables them to continually compare the working conditions and quality of life available in key destination countries, and this informs their migration decision making, as it does for other migrant health workers [16, 17]. Many successful medical doctors have ‘boundaryless global career[s]’ [18], similar to the international careers enjoyed by professional football players [19] and other elite groups of highly skilled professionals.

The tradition of doctor emigration involved doctors emigrating to obtain specialist skills and experience before returning to Ireland to take up a consultant post. However, austerity appears to have dented Ireland’s ability to compete and attract back emigrant Irish-trained doctors [3, 15], particularly at consultant level [16]. Austerity-related cuts to health spending in Ireland since 2008/2009 have reduced staffing levels and intensified long-standing (and unresolved) issues in the Irish health system—such as the need for clearer training and career progression pathways, the need to comply with the European Working Time Directive, and the need for a consultant-delivered health service [17, 20, 21]. Austerity has also resulted in a 30% reduction to consultant salaries for new entrants. As a result, ‘a full-time hospital consultant post is no longer as sought after, having previously been the pinnacle of a career, particularly for Irish born doctors who were working abroad’ [22]. The authors propose a reappraisal of the culture of medical migration, its potential impact on the dynamics of doctor emigration and return and the potentially negative impact on the medical workforce and wider health system.

## Methods

### Qualitative data collection

This paper draws on secondary quantitative and primary qualitative data generated as part of the Doctor Emigration Project, a project focussed on doctor emigration from Ireland. Research ethics approval was obtained from the RCSI Research Ethics Committee. The project drew on an initial national survey of 1636 trainee doctors in Ireland, conducted in 2014 [23]. Survey respondents were asked to participate in the qualitative component of the Doctor Emigration Project. In-depth interviews were conducted with 45 of these doctors. A further five respondents were recruited via snowball sampling. Researchers sought to include respondents who had emigrated as well as those who had stayed and to interview respondents working at different grades, in different specialties and at various geographic locations [24]. Interviewing continued until data saturation was achieved.

Of the 50 doctors interviewed, 11 had migrated, 15 had definite plans to migrate and 24 intended to remain in Ireland (at least until the completion of their postgraduate specialist training), and current migration status is reported alongside each *verbatim* quotation (Abroad, Ireland and planning to migrate, Ireland and planning to remain). To protect respondent confidentiality, respondent grades, speciality, stage of training and gender are not reported alongside quotations. The respondents ranged in age from 24 to 39, with an average age of 32. Twenty-nine respondents were female and 21 male. Forty-three of the 50 respondents had received their basic medical training in Ireland. Interviews were conducted by authors (SC and NH) between March and July 2015. Interviews were conducted face to face, by telephone or by Skype. All interviews were audio recorded and transcribed *verbatim*. All respondents consented to their participation in the research process.

Theme sheet development was informed by the relevant literature and by previous research by the authors on health worker migration [15, 25–27]. Interviews involved a discussion of respondents’ experiences of working in Ireland, their decisions to migrate from (or remain in) Ireland and the factors influencing those decisions. The interview transcripts were imported into MaxQDA. Although the analysis process is an ongoing, iterative process beginning with data collection [28] and continuing through to the write-up and publication of findings, formal coding began with a review of the transcripts by SC and NH. This review process enabled the researchers to familiarise themselves with the data and to identify emerging themes. The transcripts were reviewed line by line by both SC and NH who applied in vivo codes and analytical memos to ‘lines, paragraphs or segments that illustrate the chosen concept’ [28]. This approach ensured that the experience of the respondents remained central to the analysis process. An underlying theme, threaded throughout the interviews, was the sense that emigration was the norm for these doctors and that a decision to remain in Ireland was unusual and needed to be justified. This was true in interviews with doctors living in Ireland as well as interviews with those who had emigrated. This resonated with Connell’s research on health workers in Pacific island states [8] and informed the decision to focus this paper on the culture of medical migration. The verbatim quotations used in the paper are representative of the wider experience relayed to researchers within the in-depth interviews.

### Secondary data on doctor migration

**Fig. 1:**
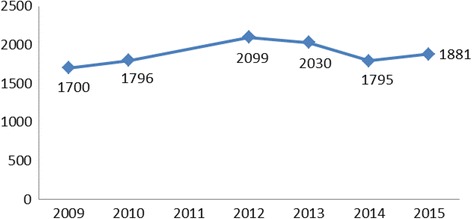
Number of verifications issued (2011 data unavailable from MCI)

**Fig. 2:**
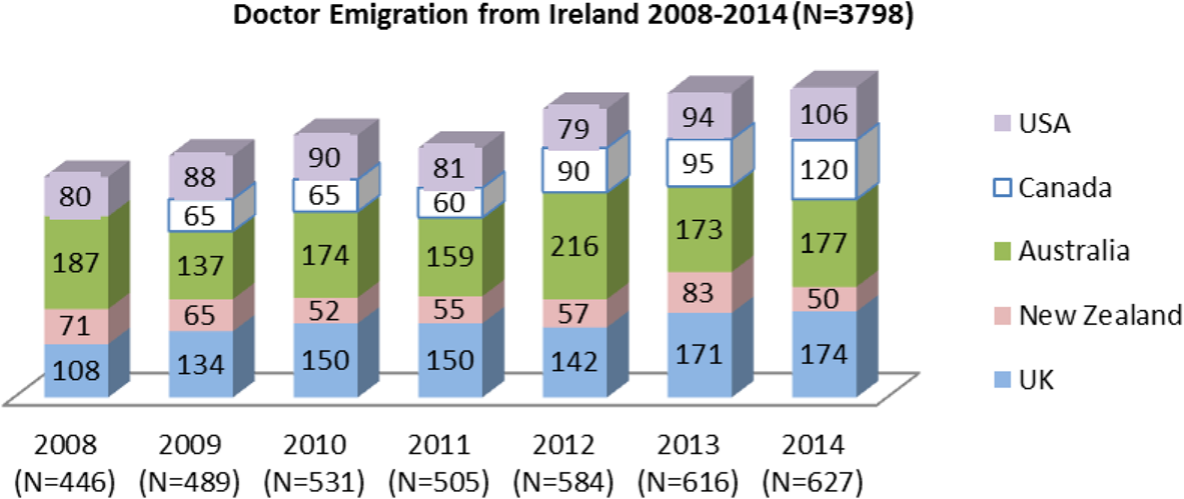
Flow of doctors from Ireland to five key destination countries in 2008–2014 (*N* = 3798)

Doctor migration from Ireland is not formally measured, but two indicators of doctor emigration are presented here (Figs 1 and 2): verification data from the source country and entry data from five key destination countries. To migrate from Ireland, doctors must have their registration on the Irish register of medical practitioners verified by the Medical Council of Ireland prior to migration. Verification data is a record of the annual number of requests processed, and the research team obtained this data for 2009–2015 from the Medical Council of Ireland. Although verification is an imperfect measure of migration in that it represents *intent* to emigrate rather than actual migration [29, 30], it is often the only available indicator of a doctor’s intention to migrate from a source country.

To obtain another measure of the number of doctors who have migrated from Ireland in recent years (see Fig. 2), the authors gathered data from five key destination countries from 2008 to 2014 for Irish-trained doctors via professional registers and/or immigration records—the UK, the USA, Canada, Australia and New Zealand. The data gathered were as follows: UK data from the General Medical Council on Irish-trained doctors joining the UK register; Australian data from the Department of Immigration and Border Protection—Irish citizen doctors issued with temporary visas; US data from the American Medical Association Masterfile (via FAIMER)—Irish resident doctors issued with J1 visas; Canada data from Citizenship and Immigration Canada—Irish citizen doctors issued with work permits; and New Zealand data from the Medical Council of New Zealand—Irish-trained doctors joining the New Zealand register). Although not a definitive or comprehensive measure of doctor migration from Ireland, it again is the best available indicator of the scale of doctor migration from Ireland in recent years, available via destination country databases.

## Results

### Scale of medical migration from Ireland

Ireland has had a long tradition of medical migration to destinations such as the UK and the USA [8, 31, 32], with doctors comprising a distinctive subgroup within a larger migration flow [33]. Although doctor migration has generally been poorly quantified, research by Oscar Gish found that 71% of Irish medical graduates in 1950–1966 migrated (cited in [34]), driven by poor medical career advancement opportunities at home and a desire for specialist training opportunities abroad [32]. As in many other European countries [35], the outward migration of doctors is poorly quantified, but the available data indicate significant levels of doctor migration. For instance, according to the HSE, more than half of the doctors who completed their internship in Ireland in 2011 had migrated within the year [36]. Figure 1 presents the overall number of verification certificates issued by the Medical Council of Ireland in 2009–2015. Although an imperfect measure of outward doctor migration (see the ‘Research methods’ section), they indicate a consistently high level of migration intent by doctors in Ireland, given that Ireland graduated 3500 Irish (and other EU) doctors during the same period.

A second measure of doctor migration from Ireland was obtained via professional registration and/or immigration data from the USA, the UK, Australia, New Zealand and Canada, key destination countries for migrant doctors (see the ‘Research methods’ section) [15, 27, 37]. Data recorded their point of entry to the destination country. The data indicate that approximately 3798 doctors migrated from Ireland to these five key destination countries between 2008 and 2014 (see Fig. 2). This represents a significant level of doctor migration. By way of context, Ireland’s six medical schools combined graduated approximately 3500 Irish/EU doctors over the same period. Despite the limitations associated with the data presented (see the ‘Limitations’ section), they indicate a trend of large-scale emigration of doctors from Ireland and underscore the need for accurate and consistent measurement of outward doctor migration.

### A culture of medical migration

Interviews revealed a strong culture of medical migration—42/50 respondents spoke about the importance of migration in ensuring career progression in medicine. According to the respondents, only a small number of specialties, such as general practice (GP), did not encourage trainees to emigrate to achieve career progression (GP respondents cited financial motivation as a driver of their migration). Five of the 50 respondents discussed emigration primarily in terms of an escape from the working conditions in the Irish health system, and a final three respondents spoke of their firm desire to remain in Ireland.

Those respondents (42/50) who discussed the importance of migration in career progression felt that international experience (either via fellowships or international experience) was essential to their medical career progression, even if their intent was ultimately to work in the Irish health system, as this respondent illustrates:

going abroad at the end of my specialist training. . . it’s something you have to do if you want to become a consultant...you go abroad for a year, you train up in something that isn’t in Ireland and you bring it back (respondent 31/Ireland and plan to remain).

The need to go abroad to acquire skills and experience of working in another health system was considered part and parcel of becoming a specialist in the Irish health system and was described by several respondents as ‘a tradition’ (respondent 10/Ireland and plan to migrate). In presenting migration as an essential component of a medical career in Ireland, the culture of medical migration compelled doctors to leave Ireland, either permanently or temporarily, regardless of their personal preferences.

I was told recently that if I didn’t go away to do a fellowship that I would not be employable in Ireland (respondent 12/Ireland and plan to migrate).

It would be almost unheard of to train exclusively in Ireland and go into a consultant post (respondent 1/Ireland and plan to migrate).

Like other small countries today [38], Ireland previously lacked the capacity for specialist postgraduate medical training and so migration was necessary for the acquisition of specialist skills. The return to Ireland of doctors with internationally acquired specialist and sub-specialist skills is generally considered to have enhanced Ireland’s specialist training capacity.

most of the consultants have either worked in the UK, Australia or Canada for a year or two and have then come back to Ireland in their post (respondent 19/Ireland and plan to migrate).

Ireland now has the capacity to provide postgraduate specialty training in almost all medical and surgical specialties, but the tradition of emigration remains. Doctors today are still expected to work in international centres of excellence in order to ‘get the cutting edge stuff’ (respondent 15/Ireland and plan to remain), improve their career prospects, or, as one respondent put it, so that ‘your CV looks flashier’ (respondent 33/Abroad). International experience and specialist training are still considered superior to anything available locally, a message similar to that conveyed to trainees in Pakistan [13] and in the Pacific islands where both ‘the workplace and. . . culture stressed that excellence was elsewhere’ [8]. In this way, migration is synonymous with ambition and medical career progression. The likelihood that doctors with international experience on their CVs would achieve career progression ahead of those without is communicated to the respondents via their interactions with interview panels, mentors and peers. In this way, the culture of migration is communicated to a new generation of doctors.

going abroad at the end of my specialist training . . . it’s something you have to do if you want to become a consultant. . . you go abroad for a year, you train up in something that isn’t in Ireland and you bring it back (respondent 31/Ireland and plan to remain).

I would find it very difficult to advance in an Irish setting without doing a fellowship abroad (respondent 13/Abroad).

Traditionally, migration and international experience were deemed necessary to differentiate the CV of the individual doctor from those of colleagues in the competition for consultant posts in the Irish health system. Lindberg highlights that when most applicants share common levels of formal education and training, ‘the ‘soft currencies’ of experience and achievements outside formal education become increasingly important’ [14].

The traditional pattern of medical migration was assumed, in the past, to be a circular one. Irish-trained doctors would emigrate and enhance their skills and experience before returning to compete for and take up consultant posts in the Irish health system. Although there were no data to track the circular component of medical migration, they were generally considered in a positive light—‘out of country experiences were perceived as strengthening the Irish medical education and health care systems’ [39]. Senior doctors returned in sufficient numbers to ensure that consultant vacancies were rare and that there was strong competition for the vacancies arising. This helps to explain the high regard for international experience within the Irish medical profession [39]. A 2008 survey of Irish hospital consultants found that 90% had completed some specialist training abroad [40, 41]. The culture of migration has seen senior doctors encourage their junior colleagues to follow the well-worn pathway of emigration to secure career progression in Ireland. In essence, doctor emigration to achieve career progression represents ‘the way things have always been done’ in the Irish health system.

However, the recession of 2008/2009 has changed the context and has triggered a consultant recruitment and retention crisis in Ireland [16]. The 30% reduction to the starting salaries of newly appointed consultants, introduced in 2012, has combined with the impact of austerity-related health cutbacks [42] on the health system, to make a consultant post in the Irish health system a less attractive option for emigrant Irish-trained doctors [22]. This respondent warns that the medical profession has yet to grasp the implications of these changes.

Irish doctors . . . are encouraged to go abroad to train . . . but I don’t even think it ever really occurs to the people encouraging us that we might not come back. So that’s the danger (respondent 1/Ireland and plan to migrate).

While there has traditionally been strong competition for consultant vacancies in the Irish health system (particularly from emigrant Irish-trained doctors), a 2015 policy report noted that 200/2933 consultant posts were vacant and that 39/149 consultant vacancies advertised in 2015 received one or no applications [41]. This suggests a disruption to the traditionally strong return flow of Irish-trained doctors applying to take up consultant posts in the Irish health system. Austerity-related salary cuts are likely to have influenced these changes. As a recent policy report outlined, since 2008/2009, consultant remuneration in Ireland has not kept pace with countries such as the USA and Australia [16], both key destinations for Irish-trained doctors. As this respondent explains, Ireland has long neglected health system reform in favour of more immediate fixes to recruitment and retention problems:

in the ‘90s . . . we threw money at consultants so they would come to Ireland, we threw money at interns so that they would continue working crap hours, but eventually the money runs out . . . What we didn’t do in the ‘90s was actually reform or change any of the working practices that were potentially going to drive people away (respondent 34/ Ireland and plan to remain).

## Discussion and Conclusions 

### Widespread doctor emigration

The widespread emigration of doctors from Ireland, is a signal of systemic problems in the Irish health system. Kapur explains that ‘any system that haemorrhages talent over the long run will struggle to survive let alone prosper’ [43]. The value of medical migration to the Irish health system has rested on the assumption that emigrant doctors will ultimately return to take up consultant posts within the Irish health system. The patterns of emigration seen since 2008 suggest that the dynamics of doctor emigration have changed. The resultant negative impacts on the Irish health system include a consultant recruitment crisis [16] and a high reliance on internationally trained doctors (who account for 38% of registered doctors [4]). These challenges to the medical workforce have occurred despite recent investment in basic medical training, the number of medical students increased by 32% 2011–2015) [44]. A high level of outward doctor migration, without return, will interfere with medical workforce planning projections and may result in doctor shortages or specialty imbalances [43, 45, 46].

Another challenge associated with doctor emigration is that it deprives organisations of those who could best help it fight its shortcomings [47]. Doctor emigration means that those who best understand the shortcomings of the Irish health system and who might be well placed to challenge and improve that system, emigrate. In this way, the health system is deprived of potential leaders who might otherwise demand, initiate and deliver reform. This is a theme considered by Kapur in an Indian context [43]. Doctors who migrate but remain committed to influencing change in the Irish health system [3] are potentially a valuable resource for the health system, albeit one which has been under-utilised to date. A high-profile illustration of diaspora contribution to the Irish health system is Professor Tom Keane, an Irish-trained doctor in Canada who returned to Ireland in 2008–2010 to direct the National Cancer Control Programme and who subsequently chaired a 2016 RCPI report on the future direction of Irish healthcare [48]. There is potential for a greater level of involvement by the Irish medical diaspora, in the Irish health system. A caveat is that, despite its potential value to the source country health system, diaspora input is not a solution to doctor emigration because a few weeks per year, or even a few years within a career, does not equate to a full-time doctor working in the health system for their entire career. Eyal and Hurst discuss this in their 2008 paper, noting that diaspora input, while helpful, is not an absolute solution for the health system of the source country [45].

### Quick fixes or genuine health system reform

Although the Irish health system has adapted to high levels of doctor emigration, a reliance on internationally trained doctors to fill the gaps has been the way in which this was achieved. As the authors have previously highlighted, the emphasis has been ‘on replacing health workers, rather than changing the conditions within which they are dissatisfied’ [26]. Although replacing emigrant doctors with internationally trained doctors fills vacant posts, it also means solving Ireland’s workforce problems at the expense of another, usually poorer, country. Although this provides a temporary solution, it is unlikely to be sustainable. Indeed, genuine reform is required to encourage more Irish-trained doctors to remain working in the Irish health system and to discourage them from wanting to emigrate. As Eyal and Hurst highlight, encouraging emigrant doctors to return to their source country is always likely to pose a greater challenge than retention because ‘the same factors that motivate physicians to seek greener pastures will keep the majority there’ [45]. In a recent survey of emigrant Irish health workers (*N* = 388), only 29% intended to return to Ireland in the future, with another 29% open to the possibility [3]. The longer that health professionals remain abroad, the less likely they are to return to the source country [15, 46]. The respondents spoke about reform and improved working conditions as prerequisites for their return [3]. To encourage doctor retention and encourage the return of those who have already left, there is a need for genuine health system reform rather than quick fixes. Ireland must address the concerns of the medical workforce [3], particularly in the hospital sector.

### Encourage retention as an option

The culture of medical migration is at odds with the national workforce policy of medical workforce self-sufficiency and with the staffing needs of the Irish health system. In order to train and retain enough doctors to meet demand, there is a need to better align medical career progression pathways with future workforce needs. This will require a reappraisal of the professional culture of medical migration. Although emigration is of value within a medical career, medical students and doctors-in-training must also be assured of their future place within the Irish health system. Career progression pathways that do not necessitate emigration should be developed and promoted. Those careers or specialties that continue to require emigration should offer more structured options to their trainees, to encourage circular migration (i.e., emigration and return). The medical profession could encourage retention by assisting early career doctors to design career pathways that align with the workforce needs of the Irish health system [49] and by lobbying to ensure that the Irish health system offers ‘better working conditions, clear progression pathways and a better practice environment’ [3] to its doctors.

### Better understanding of migration dynamics

Routine data on outward and return doctor migration is ‘a prerequisite to a better understanding of health professional emigration’ [3], without which, policy makers are operating in the dark. Better routine data on doctor migration is critical and is key to capturing an accurate understanding of the dynamics of doctor emigration and return/non-return in the Irish context. It is also essential for enabling the system to measure the effectiveness of any retention measures introduced. This paper indicates that austerity has changed the extent to which emigrant doctors return to take up consultant posts in the Irish health system. Better routine data, combined with research on health worker emigration [3, 15, 23] will enable policy makers to assess the impact of austerity on doctor emigration and return and empower them to respond accordingly. It will also help them to measure the impact of other external shocks on the Irish medical workforce (e.g., the impact of Brexit [50] on doctor migration flows) and on the dynamics of doctor migration. In conclusion, there is a need to reappraise the ‘well entrenched. . . culture of migration’ [8] and its promotion of doctor migration. It’s imperative to find ways to encourage and reward Irish-trained doctors for contributing to the Irish health system, rather than encouraging and rewarding them for exiting it.

### Limitations

- In Fig. 1, the data presented indicate the overall number of verification certificates issued to doctors, rather than the number issued to an individual doctor. The data relating to 2011 was unavailable from the Medical Council of Ireland.
- In Fig. 2, the destination country data are an imperfect measure of outward doctor migration, firstly because obtaining a work permit or registration in a specific country does not necessarily equate to migration. Secondly, some countries record citizenship of the doctors entering the country, while others record their country of medical training. This means that some non-Irish nationals who trained in Ireland and/or non-Irish trained doctors who hold Irish citizenship may be included in the figures presented.
- Intern migration intention data from [36] may be confounded by the tradition of doctors taking a gap year abroad in between completion of internship and beginning of basic specialist training.
